# LXR Suppresses Inflammatory Gene Expression and Neutrophil Migration through *cis*-Repression and Cholesterol Efflux

**DOI:** 10.1016/j.celrep.2018.11.100

**Published:** 2018-12-26

**Authors:** David G. Thomas, Amanda C. Doran, Panagiotis Fotakis, Marit Westerterp, Per Antonson, Hui Jiang, Xian-Cheng Jiang, Jan-Åke Gustafsson, Ira Tabas, Alan R. Tall

**Affiliations:** 1 Division of Molecular Medicine, Department of Medicine, Columbia University, New York, NY 10032, USA; 2 Department of Pediatrics, Section Molecular Genetics, University Medical Center Groningen, University of Groningen, 9713 AV Groningen, the Netherlands; 3 Department of Biosciences and Nutrition, Karolinska Institutet, 141 83 Huddinge, Sweden; 4 Department of Cell Biology, SUNY Downstate Medical Center, Brooklyn, NY 11209, USA; 5 Center for Nuclear Receptors and Cell Signaling, University of Houston, Houston, TX 77204, USA; 6 Departments of Physiology and Pathology & Cell Biology, Columbia University, New York, NY 10032, USA; 7 Lead Contact

## Abstract

The activation of liver X receptor (LXR) promotes cholesterol efflux and repression of inflammatory genes with anti-atherogenic consequences. The mechanisms underlying the repressive activity of LXR are controversial and have been attributed to cholesterol efflux or to transrepression of activator protein-1 (AP-1) activity. Here, we find that cholesterol efflux contributes to LXR repression, while the direct repressive functions of LXR also play a key role but are independent of AP-1. We use assay for transposase-accessible chromatin using sequencing (ATAC-seq) to show that LXR reduces chromatin accessibility in *cis* at inflammatory gene enhancers containing LXR binding sites. Targets of this repressive activity are associated with leukocyte adhesion and neutrophil migration, and LXR agonist treatment suppresses neutrophil recruitment in a mouse model of sterile peritonitis. These studies suggest a model of repression in which liganded LXR binds in *cis* to canonical nuclear receptor binding sites and represses pro-atherogenic leukocyte functions in tandem with the induction of LXR targets mediating cholesterol efflux.

## In Brief

Thomas et al. show the roles of cholesterol efflux and direct repression in anti-inflammatory effects of LXR and establish the mechanism of LXR *cis*-repression using ATAC-seq. LXR agonists suppress neutrophil migration genes and neutrophil recruitment during inflammation, highlighting a potential role for these compounds in the control of neutrophil-predominant inflammatory conditions.

## Graphical Abstract

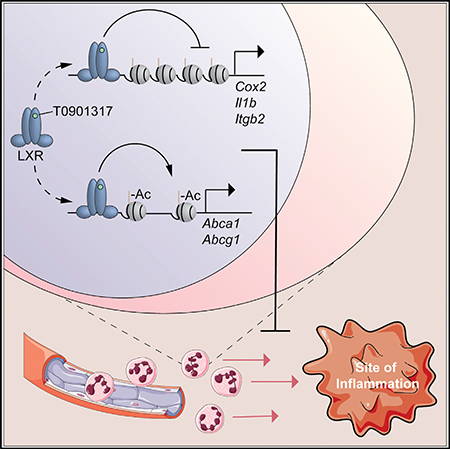

## INTRODUCTION

Macrophage inflammatory and metabolic processes determine the progression of several inflammatory diseases, such as atherosclerosis (Murray and Wynn, 2011). During atherogenesis, macrophages in the artery wall become cholesterol loaded and produce inflammatory cytokines, leading to leukocyte recruitment and plaque destabilization (Moore and Tabas, 2011). The liver X receptor (LXR) is a nuclear receptor with two isoforms, LXRα and LXRβ, that respond to oxysterols generated during cellular cholesterol loading by promoting cholesterol efflux and inflammatory gene repression (Schulman, 2017). LXR agonists are potently anti-inflammatory and anti-atherogenic in mouse models (Joseph et al., 2002, 2003).

Systemic LXR activation leads to induction of hepatic lipogenesis, prompting a search for specific anti-atherogenic functions of LXR that can be dissociated from the hepatotoxic effects of LXR activators (Schulman, 2017). Thus, it has been reported that LXR protects from atherogenesis both through induction of cholesterol efflux transporters and through transrepression of macrophage inflammatory genes (Kappus et al., 2014). LXR’s repressive activity has been attributed to the formation of a complex containing small ubiquitin-like modifier (SUMO)-modified LXR and the corepressor nuclear corepressor (NCoR) with affinity for the inflammatory transcription factor activator protein-1 (AP-1) (Ghisletti et al., 2007, 2009). Alternatively, it has been reported that LXR SUMOylation and NCoR may be dispensable for gene repression by LXR, and certain anti-inflammatory activities of LXR may reflect the metabolic functions of LXR targets, including the cholesterol efflux transporter ATP-binding cassette transporter A1 (ABCA1) (Ito et al., 2015).

Here, we use genetic and pharmacological models to establish that LXR repression is only partly dependent on cholesterol efflux and independent of AP-1 transactivation. Rather, based on studies using RNA sequencing (RNA-seq), assay for transposase-accessible chromatin using sequencing (ATAC-seq), and alignment of LXR chromatin immunoprecipitation sequencing (ChIP-seq) with ATAC-seq data, the direct repressive function of LXR appears to be mediated through *cis*-binding of LXR to enhancer elements, leading to chromatin closure. LXR repression specifically regulates a subset of genes comprising chemokines and adhesion molecules involved in regulating neutrophil migration in the setting of low-grade inflammation. We demonstrate that LXR agonist treatment attenuates neutrophil migration during sterile inflammation *in vivo*, which is associated with LXR *cis*-repression and regulation of cholesterol metabolism in a cell-intrinsic manner. Thus, LXR regulates inflammation and neutrophil migration through both metabolic and repressive functions.

## RESULTS

### Cholesterol Efflux Transporters Partly Mediate LXR Repression

To study mechanisms of inflammatory gene repression by LXR, we used bone-marrow-derived macrophages (BMDMs) treated with the inflammatory Toll-like receptor 4 (TLR4) agonist lipopolysaccharide (LPS) and the LXR agonist T0901317 (T0) and genetically or pharmacologically perturbed potential mediators of LXR functions. LXR activation by T0 for 3 hr prior to LPS stimulation is associated with repression of the inflammatory genes *Cox2* and *Il1b* (Figure S1A). This repressive effect of T0 is lost at high doses of LPS (Figure S1B). T0 activates LXR as well as FXR and PXR in hepatocytes and ROR-γt in T cells (Houck et al., 2004; Mitro et al., 2007; Solt et al., 2012). However, the anti-inflammatory activity of T0 in macrophages is abrogated by knockout of LXRα and LXRβ in macrophages, demonstrating specificity for LXR (Figure S1A).

**Figure 1.**
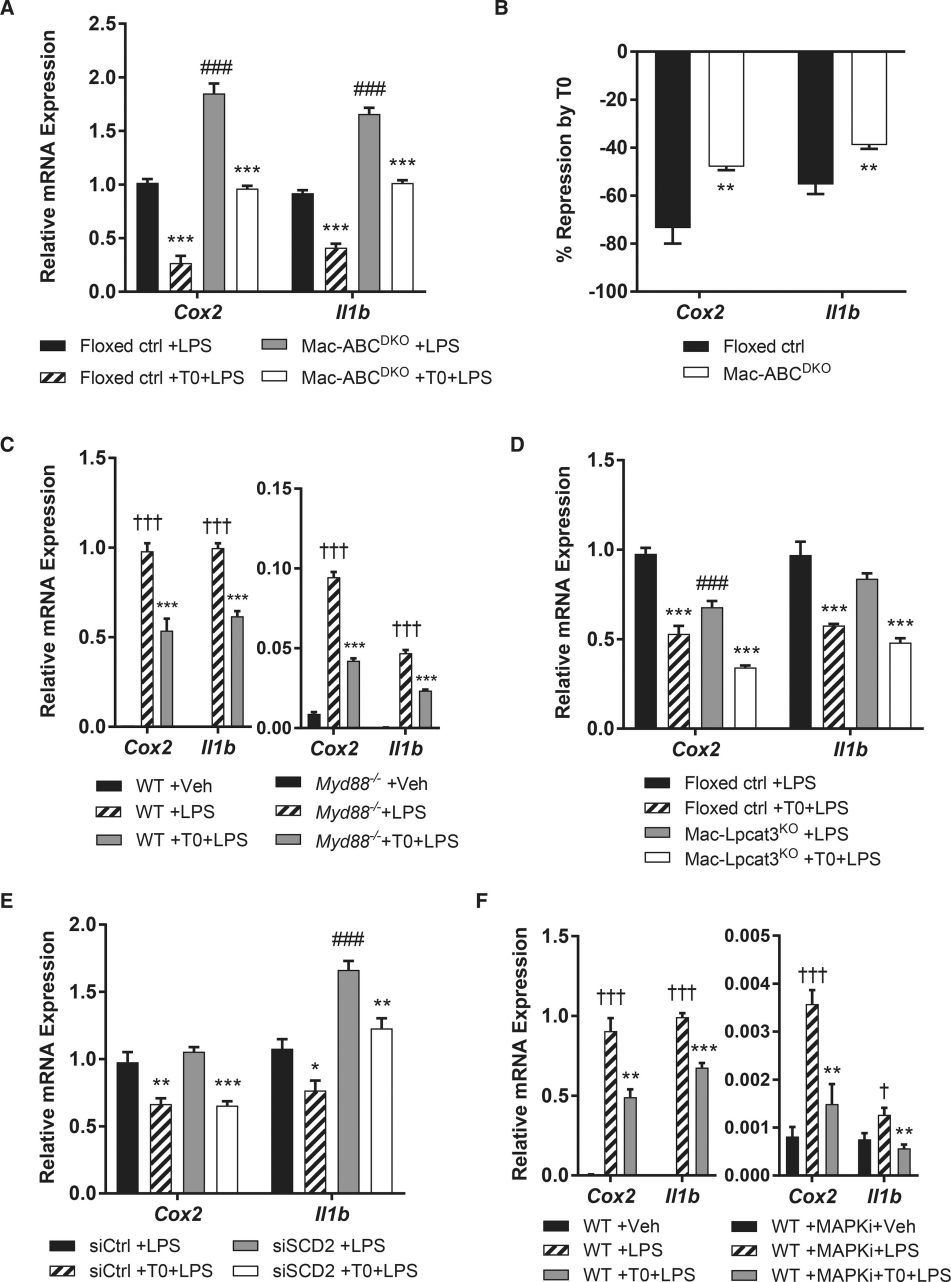
Cholesterol Efflux Transporters Partly Mediate LXR Repression (A) *Abca1*^*fl/fl*^*Abcg1*^*fl/fl*^ (floxed ctrl) or *LysMCre Abca1*^*fl/fl*^*Abcg1*^*fl/fl*^ (Mac-ABC^DKO^) BMDMs were treated for 3 hr with 500 nM T0 before stimulation with 10 ng/mL LPS for 2 hr. (B) Data in (A) plotted as percent repression normalized to the extent of LPS-inducible gene expression in each genotype. (C) Wild-type (WT) or *Myd88*^–/–^ BMDMs were treated as in (A). (D) *Lpcat3*^*fl/fl*^ (floxed ctrl) or *LysMCre Lpcat3*^*fl/fl*^ (Mac-Lpcat3^KO^) BMDMs were treated as in (A). (E) BMDMs were transfected with siSCD2 SMARTpool siRNA or non-targeting siRNA (siCtrl) for 24 hr, rested for 24 hr, and then treated as in (A). (F) BMDM were treated with MAPK inhibitors (10 μM PD0325901 and 1 μM BIRB0796) and then treated as in (A). mRNA expression was evaluated by qPCR, and mean ± SEM is plotted. n = 4 biological replicates. Significance was determined by one-way ANOVA with Tukey’s post hoc test (A and C–F) or Student’s t test with Benjamini-Hochberg multiple testing correction (B). *p < 0.05, **p < 0.01, and ***p < 0.001 for T0 treatment versus control; #p < 0.05, ##p < 0.01, and ###p < 0.001 for alternative genotype; †p < 0.05, ††p < 0.01, and †††p < 0.001 for LPS versus vehicle (Veh). Data are representative of two independent experiments. See also Figure S1.

We used this model to examine the requirement for LXR targets in inflammatory gene repression by LXR. The LXR target ABCA1 has been reported to antagonize TLR4 signaling by interfering with its adaptor protein, MYD88 (Ito et al., 2015). Consistent with earlier studies, we found that knockout of *Abca1* and the related cholesterol efflux transporter *Abcg1* in macrophages partly but significantly attenuates the anti-inflammatory effect of LXR agonists (Figures 1A and 1B). We used *Myd88* knockout macrophages to assess the requirement for this potential target of cholesterol depletion in the repressive effect of T0. Knockout of *Myd88* is associated with reduced expression of *Cox2* and *Il1b* (Figure 1C), which may alter mechanisms of inflammatory gene induction. With this caveat, we observed repression of *Cox2* and *Il1b* by T0 in these macrophages, suggesting a MYD88-independent effect (Figure 1C). In sum, cholesterol efflux transporters induced by LXR appear to play a role in LXR repression in addition to other activities of LXR or LXR targets.

Other LXR targets involved in metabolism, such as lysophosphatidylcholine acyltransferase 3 (*Lpcat3*), stearoyl-CoA desaturase 2 (*Scd2*), and the efferocytosis receptor *Mertk*, have also been linked to anti-inflammatory effects in various models (A-Gonzalez et al., 2009; Li et al., 2013; Rong et al., 2013). We addressed whether they play a role in LXR repression in macrophages using loss-of-function approaches. Knockout of *Lpcat3* in macrophages reduces phosphatidylcholine polyunsaturated fatty acid (PUFA) content (Figure S1C) but has no effect on LXR repression of *Cox2* and *Il1b* (Figure 1D). Likewise, knockdown of *Scd2* by small interfering RNA (siRNA) does not affect LXR repression (Figures 1E and S1D). MERTK blockade using a neutralizing antibody approach (Sen et al., 2007) similarly has no effect on repressive effects of the LXR agonist T0 in macrophages (Figure S1E).

These findings suggest a potential role for LXR in direct repression of inflammatory gene enhancers, as reported in earlier studies in which LXR was proposed to interfere with AP-1 transactivation (Ghisletti et al., 2009). Thus, we inhibited LPS-inducible AP-1 activation using inhibitors of mitogen-activated protein kinases (MAPKs) (Tong et al., 2016), which phosphorylate and activate AP-1 (Kawai and Akira, 2010). MAPK inhibitor treatment markedly reduces *Cox2* and *Il1b* expression, consistent with defective AP-1 activation (Figure 1F). With the caveat that gene expression is markedly reduced in this setting, *Cox2* and *Il1b* remain LPS inducible and repressed by T0 (Figure 1F). Together, these observations suggest that direct repression by LXR may be independent of MAPK and AP-1 signaling, which is inconsistent with existing mechanistic models (Glass and Saijo, 2010).

### LXR Agonist Closes Chromatin at Inflammatory Gene Enhancers

To further explore potential mechanisms responsible for LXR repression of inflammatory genes, we assessed the identity of gene regulatory elements associated with inflammatory gene repression using ATAC-seq. ATAC-seq identifies genomic regions susceptible to DNA insertion by Tn5 transposase, and these regions are associated with gene-regulatory activity (Buenrostro et al., 2013). Using ATAC-seq, we determined open chromatin regions in control macrophages or macrophages treated with T0, LPS, or both (Table S1). We focused our analysis on macrophage enhancers, defined by activating histone marks H3K27ac or H3K4me2 in published primary macrophage ChIP-seq data (Oishi et al., 2017). Enhancer filtering captured 70% of open chromatin regions and limited our analysis to regions likely to be involved in macrophage gene expression (Lavin et al., 2014). Principal component analysis of the ATAC signal over all macrophage enhancers showed that replicates cluster by condition, establishing the reproducibility of the assay, with a strong effect of LPS and a moderate effect of T0 in the first two principal components (Figure S2A).

**Figure 2.**
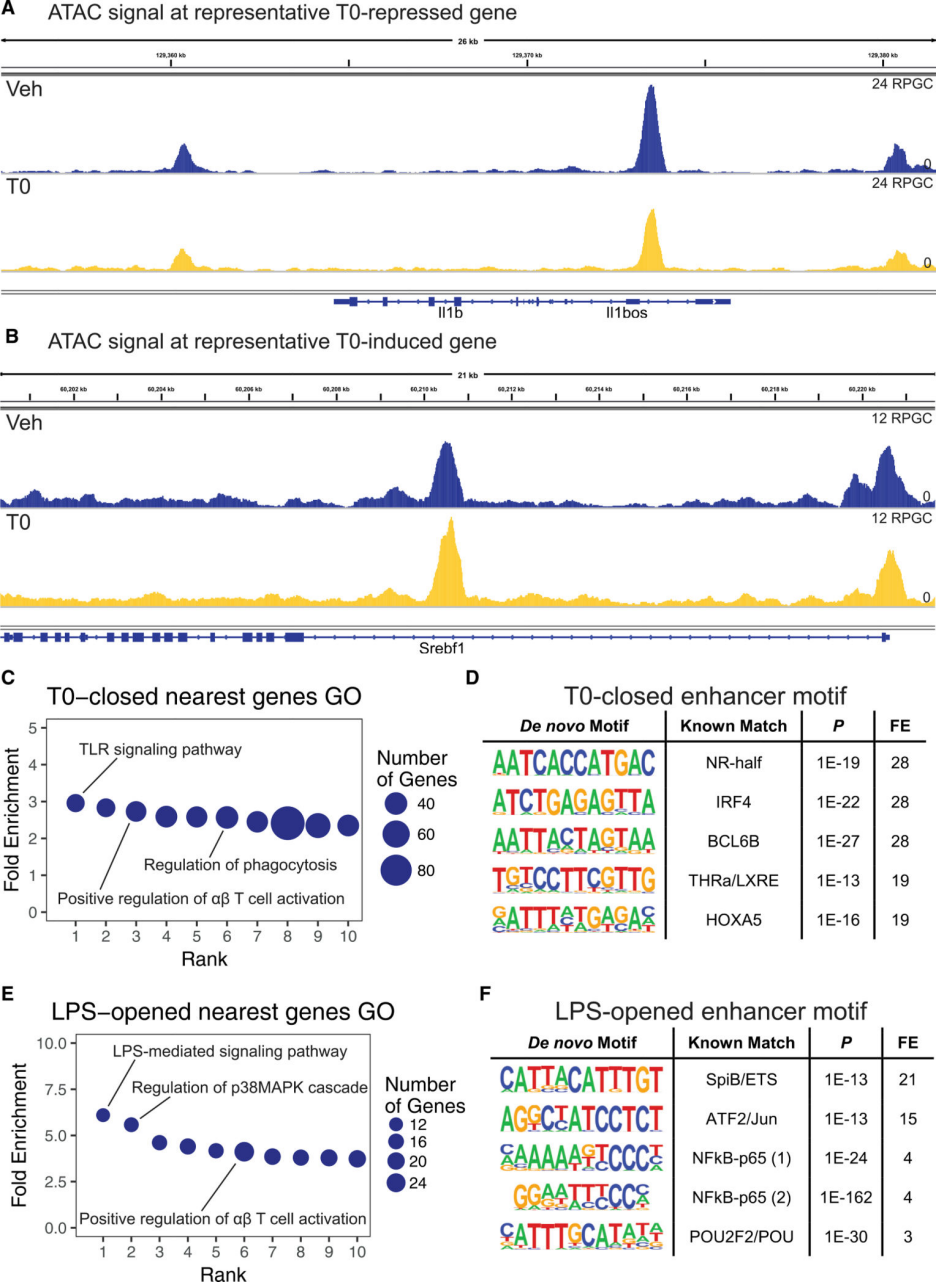
LXR Agonist Closes Chromatin at Inflammatory Gene Enhancers WT BMDMs were treated for 3 hr with 500 nM T0 before stimulation with 10 ng/mL LPS for 2 hr and harvested for ATAC-seq. Accessible regions were determined from ATAC-seq data, and analysis was restricted to macrophage H3K4me2- or H3K27ac-marked enhancers defined by Oishi et al. (2017). (A and B) Representative genome browser track of chromatin accessibility signal around the T0-repressed gene *Il1b* (A) and the T0-induced gene *Srebf1* (B) with vehicle or T0 treatment. Signal is plotted in units of reads per genomic content (RPGC). (C) PANTHER GO categories enriched in genes nearest to T0-closed enhancers (Bonferroni-adjusted p < 0.05). (D) HOMER de *novo* motifs enriched in T0-closed enhancers (p < 1 3 10^–12^; top 5 motifs displayed). FE, fold enrichment; LXRE, LXR response element; NR-half, nuclear receptor half-site. (E) PANTHER GO categories enriched in genes nearest to LPS-opened enhancers (Bonferroni-adjusted p < 0.05). (F) HOMER de *novo* motifs enriched in LPS-opened enhancers (p < 1 3 10^–12^; top 5 motifs displayed). n = 4 biological replicates. FE, fold enrichment. See also Figure S2 and Tables S1–S5.

T0 treatment leads to decreases in chromatin accessibility at targets of LXR repression, such as *Il1b* (Figure 2A), and moderate increases in chromatin accessibility at LXR targets, such as *Srebf1* (Figure 2B). On a genome-wide basis, T0 treatment is associated with closure of 6,474 enhancers (“T0-closed”) and opening of 224 enhancers (“T0-opened”) (Figure S2B). We used Gene Ontology (GO) analysis to assess cellular functions that may be regulated by each enhancer set by determining the gene nearest to each enhancer and performing GO analysis (Thomas et al., 2003) on the corresponding gene set. T0-closed enhancers are nearest to genes associated with GO categories related to TLR signaling, positive regulation of T cell activation, and regulation of phagocytosis, linking the observed chromatin closure events to LXR’s anti-inflammatory activity (Figure 2C; Table S2). Sequence motif analysis of T0-closed enhancers using HOMER (Heinz et al., 2010) revealed an enrichment for nuclear receptor half-site motifs and direct repeat-4 (DR4) LXR response elements (Figure 2D; Table S3), suggesting that LXR may bind directly at these enhancers. Analysis of enhancers closed by T0 after LPS treatment (“T0-closed-in-LPS”), 36% of which overlap with T0-closed enhancers, confirmed the observed associations with inflammatory gene GO categories and nuclear receptor response element motifs (Figures S2C and S2D).

To validate our enhancer identification method, we examined the effect of LPS treatment. LPS treatment led to the opening of 2,020 enhancers (“LPS-opened”) and closure of 4,636 enhancers (“LPS-closed”) (Figure S2B). Genes nearest to LPS-opened enhancers are linked to GO categories related to LPS-mediated signaling and positive regulation of T cell activation, which are highly similar to the GO categories of genes nearest to T0-closed enhancers (Figure 2E; Table S4). HOMER motif analysis of LPS-opened enhancers revealed a prominent enrichment for nuclear factor-κB (NF-κB) and AP-1 binding sites (Figure 2F; Table S5), consistent with previous studies of inflammatory enhancers (Tong et al., 2016). In untreated or TLR4-stimulated macrophages, the ATAC signal correlates strongly with H3K27 acetylation signal measured in published primary macrophage ChIP-seq data (Oishi et al., 2017) (r = 0.8 for each condition; Figures S2E and S2F) and captures 80% of H3K27ac+ peaks, consistent with prior evidence that chromatin accessibility changes are linked to histone modification changes involved in transcriptional regulation (Bell et al., 2011; Mueller et al., 2017).

### LXR Binding by ChIP-Seq Localizes at T0-Closed Enhancers

**Figure 3.**
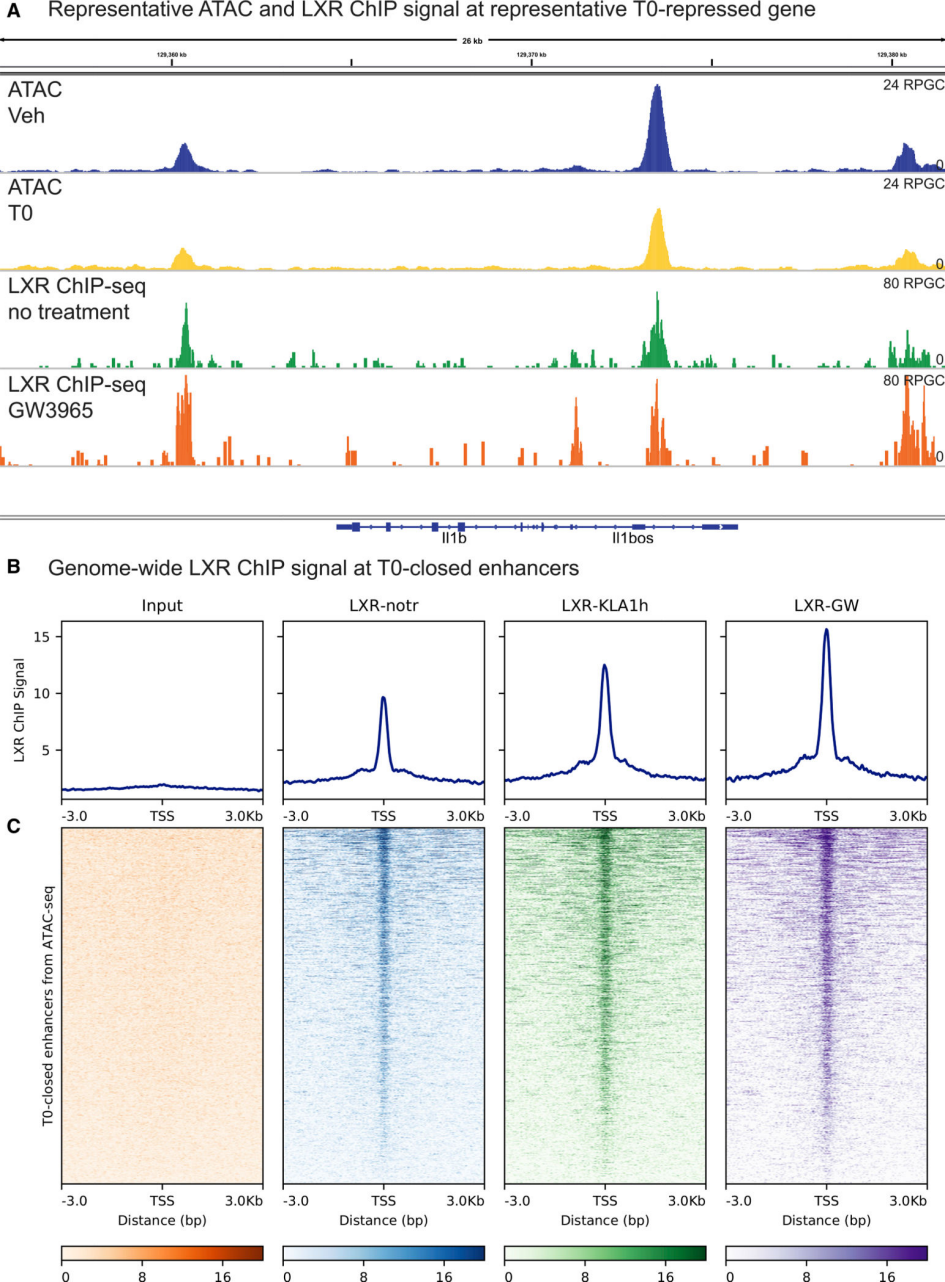
LXR Binding by ChIP-Seq Localizes at T0-Closed Enhancers The LXR ChIP-seq signal from Oishi et al. (2017) was plotted at enhancer sets derived from ATAC-seq of BMDMs treated for 3 hr with 500 nM T0 before stimulation with 10 ng/mL LPS for 2 hr. (A) Representative genome browser track of the LXR ChIP signal around the T0-repressed gene *Il1b*. The signal is plotted in units of reads per genomic content (RPGC). (B) Histogram of the LXR ChIP-seq signal centered on T0-closed enhancers. LXR-GW, chromatin from thioglycolate-elicited macrophages (TGEMs) treated with the LXR agonist GW3965 for 24 hr; LXR-KLA1h, chromatin from TGEMs stimulated with the TLR4 agonist KLA for 1 hr; LXR-notr, chromatin from resting TGEMs immunoprecipitated with anti-LXR antibody (notr, no treatment). (C) Heatmap of the LXR ChIP-seq signal as in (B) centered on T0-closed enhancers. n = 4 biological replicates (ATAC-seq) or 1 biological replicate (LXR ChIP-seq). See also Figure S3.

Motif analysis of T0-closed enhancers suggested that LXR may bind directly at these sites. To further assess this possibility, we aligned the LXR ChIP signal from a published dataset (Oishi et al., 2017) to enhancers closed or opened by T0 from our ATAC-seq data. In this published LXR ChIP-seq dataset, the LXR agonist GW3965 was used to activate LXR; the specificity for LXR binding was established at the level of antibody recognition of LXR and confirmed by motif enrichment (Oishi et al., 2017). LXR ChIP peaks are closely aligned with regions where T0 treatment reduced chromatin accessibility, as at *Il1b* (Figure 3A). On a genome-wide basis, the LXR ChIP signal is superimposed on T0-closed enhancers, producing a single peak of the LXR ChIP signal in histograms centered on closed enhancers (Figure 3B). Plotting the individual LXR ChIP signals for each enhancer after aligning the enhancer centers showed that LXR binding is present at many enhancers before LXR activation or TLR4 stimulation by the agonist Kdo2-lipid A (KLA) and at most enhancers after LXR or TLR4 stimulation (Figure 3C).

Alignment of LXR ChIP-seq to T0-closed-in-LPS enhancers revealed that LXR ChIP signal localized to these regions as well (Figures S3A and S3B). The LXR ChIP signal intensity is similar between T0-closed and T0-closed-in-LPS enhancers, suggesting a common mechanism of LXR recruitment in the presence or absence of inflammatory stimulation. At T0-opened enhancers, the LXR ChIP signal is similarly enriched (Figures S3C and S3D), consistent with the established *cis*-activating activity of LXR at enhancers of LXR targets. GW3965 treatment increased the LXR ChIP signal at enhancers closed by T0 (Figure 3B), consistent with previous observations that DNA-binding affinity of LXR is increased with LXR agonist treatment (Pehkonen et al., 2012). In combination with the enrichment for LXR binding sites in T0-closed enhancers, the alignment of LXR ChIP signal with these sites indicates that LXR binds in *cis* to enhancers of inflammatory genes.

### Chromatin Accessibility Changes with T0 Are Linked to Gene Expression Changes

**Figure 4.**
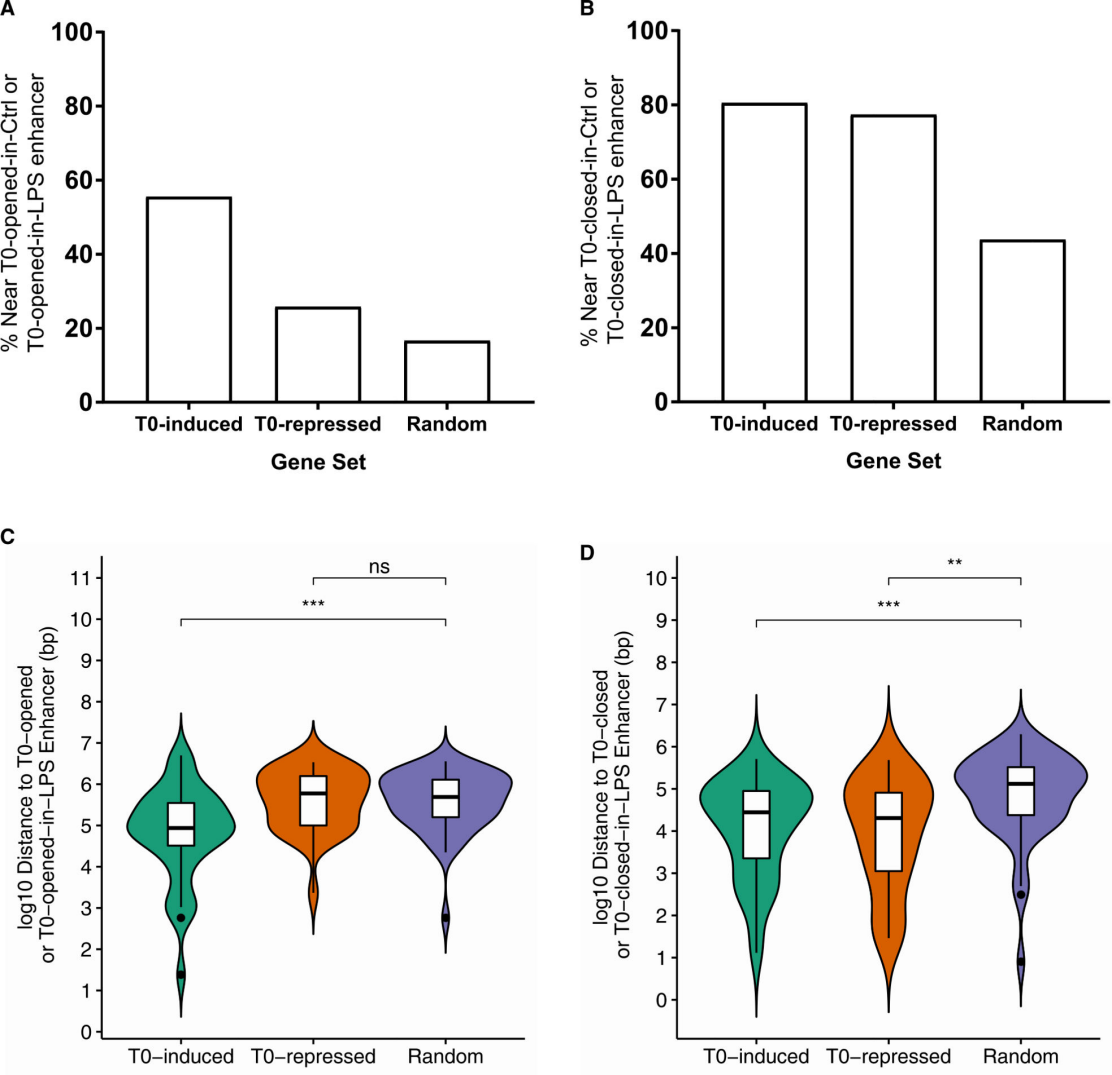
Chromatin Accessibility Changes with T0 Are Linked to Gene Expression Changes Transcription start site (TSS) positions of differentially expressed genes in RNA-seq of BMDMs treated with T0 for 3 hr and 10 ng/mL LPS for 2 hr were compared to positions of enhancers opened or closed by T0 in ATAC-seq data collected from BMDMs in the same conditions. (A and B) Percentage of genes in T0-induced, T0-repressed, or random genes with an enhancer opened by T0 (A) or closed by T0 (B) in the control or LPS-stimulated condition within 100 kb of the TSS. (C and D) Distribution of distances from the TSS to the nearest enhancer for T0-induced, T0-repressed, or random genes to enhancers opened by T0 (C) or closed by T0 (D) in the control or LPS-stimulated condition. n = 4 biological replicates (ATAC-seq) or 3 biological replicates (RNA-seq). Significance was determined by Kruskal-Wallis nonparametric one-way ANOVA with Dunn post hoc test. *p < 0.05, **p < 0.01, ***p < 0.001; ns, not significant.

We used RNA-seq to determine the functional correlates of T0-associated changes in chromatin accessibility at the level of gene expression. We treated BMDMs with or without T0 for 3 hr before stimulation with 10 ng/mL LPS for 2 hr, conditions identical to those in which we established that *Cox2* and *Il1b* repression by T0 is LXR-dependent. Comparison of genes and enhancers regulated by T0 revealed that 56% of >1.5-fold T0-induced genes are associated with a T0-opened or T0-opened-in-LPS enhancer within 100 kb of the transcription start site (Figure 4A), while 78% of >1.5-fold T0-repressed genes are associated with a T0-closed or T0-closed-in-LPS enhancer (Figure 4B). These associations are significant compared with randomly selected genes when the enhancer-promoter distance distributions are systematically compared (Figures 4C and 4D), as expected based on the association of chromatin accessibility with gene regulatory activity. Unexpectedly, 81% of T0-induced genes are also associated with either T0-closed or T0-closed-in-LPS enhancers (Figure 4B), suggesting that *cis*-binding of LXRs near these genes leads to both chromatin opening and closure events. Thus, the proximity of enhancers regulated by T0 to T0-induced or T0-repressed genes provides evidence that the observed accessibility changes correlate with transcriptional regulation events and supports our observation that LXR binding leads to chromatin closure at many sites.

### LXR Represses Neutrophil Migration Genes

**Figure 5.**
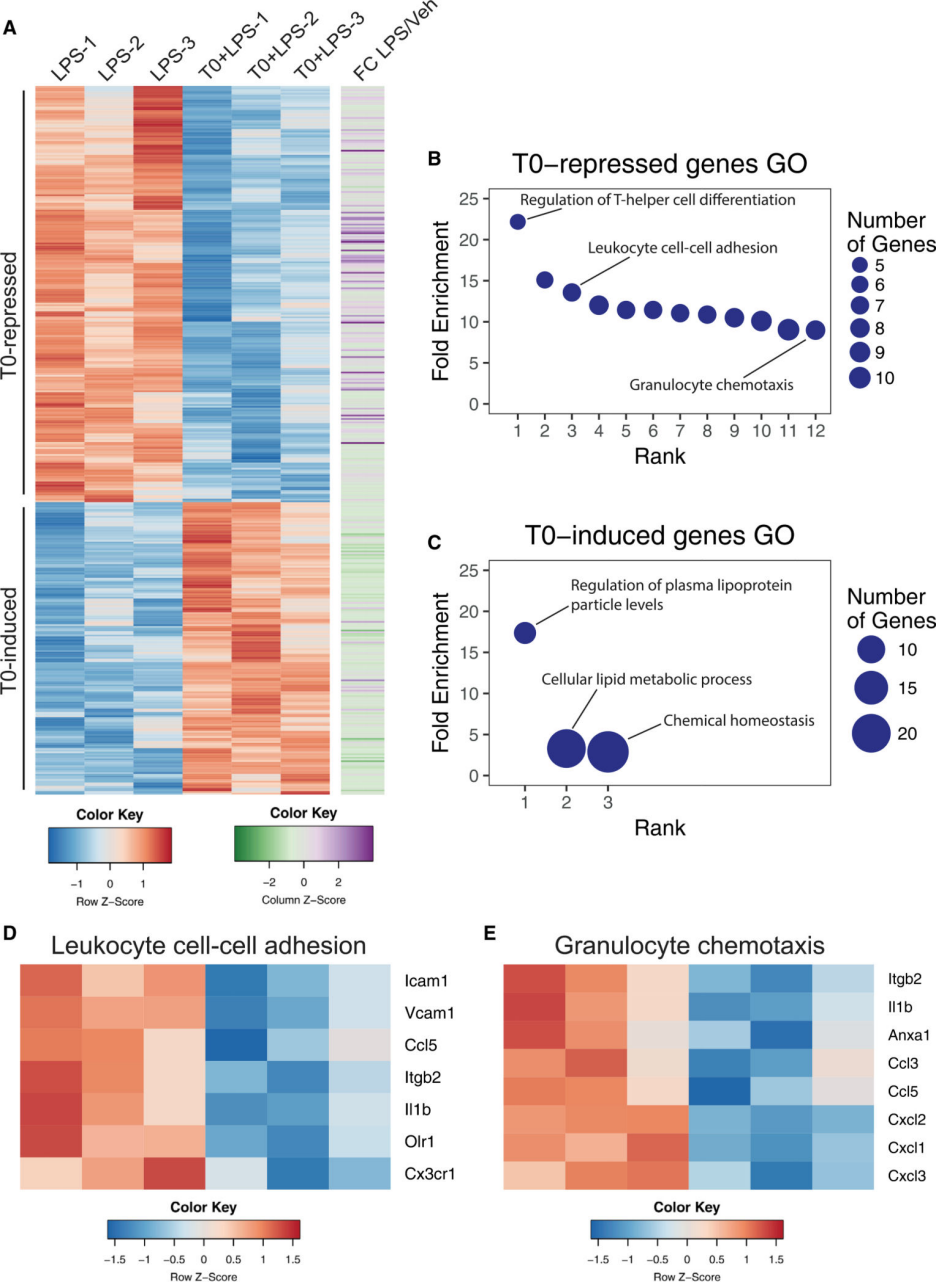
LXR Represses Neutrophil Migration Genes WT BMDMs were treated for 3 hr with 500 nM T0 before stimulation with 10 ng/mL LPS for 2 hr and harvested for RNA-seq. (A) Heatmap of all induced or repressed genes at 5% FDR, colored by row-normalized *Z* score, with extent of induction by LPS indicated on right. (B) PANTHER GO categories enriched in T0-repressed genes (Bonferroni-adjusted p < 0.05). (C) PANTHER GO categories enriched in T0-induced genes (Bonferroni-adjusted p < 0.05). (D and E) Row-normalized Z score for T0-repressed genes in the GO category “Leukocyte cell-cell adhesion” (D) or “Granulocyte chemotaxis” (E). n = 3 biological replicates. See also Table S6.

In total, T0 treatment represses 242 genes and induces 170 genes at a false discovery rate (FDR) threshold of 5% (Figure 5A). More than half of T0-repressed genes (61%) are LPS-inducible inflammatory genes (Figure 5A). GO analysis showed that T0-repressed genes are associated with immune-related functions, including regulation of T-helper cell differentiation, leukocyte cell-cell adhesion, chemokine signaling, and granulocyte chemotaxis (Figure 5B; Table S6). T0-induced genes are associated with lipoprotein activity and cellular lipid metabolism (Figure 5C). As the roles of LXR in lipid metabolism and control of T cell priming are well established (Ito et al., 2016), we investigated the enrichment of multiple GO categories regulating leukocyte and specifically neutrophil migration among T0-repressed genes. Leukocyte cell-cell adhesion and granulocyte chemotaxis genes repressed by T0 include the cytokine *Il1b*, chemokines, and adhesion molecules, including the beta integrin gene *Itgb2* (Figures 5D and 5E).

### LXR Activation Suppresses Neutrophil Migration *In Vivo*

**Figure 6.**
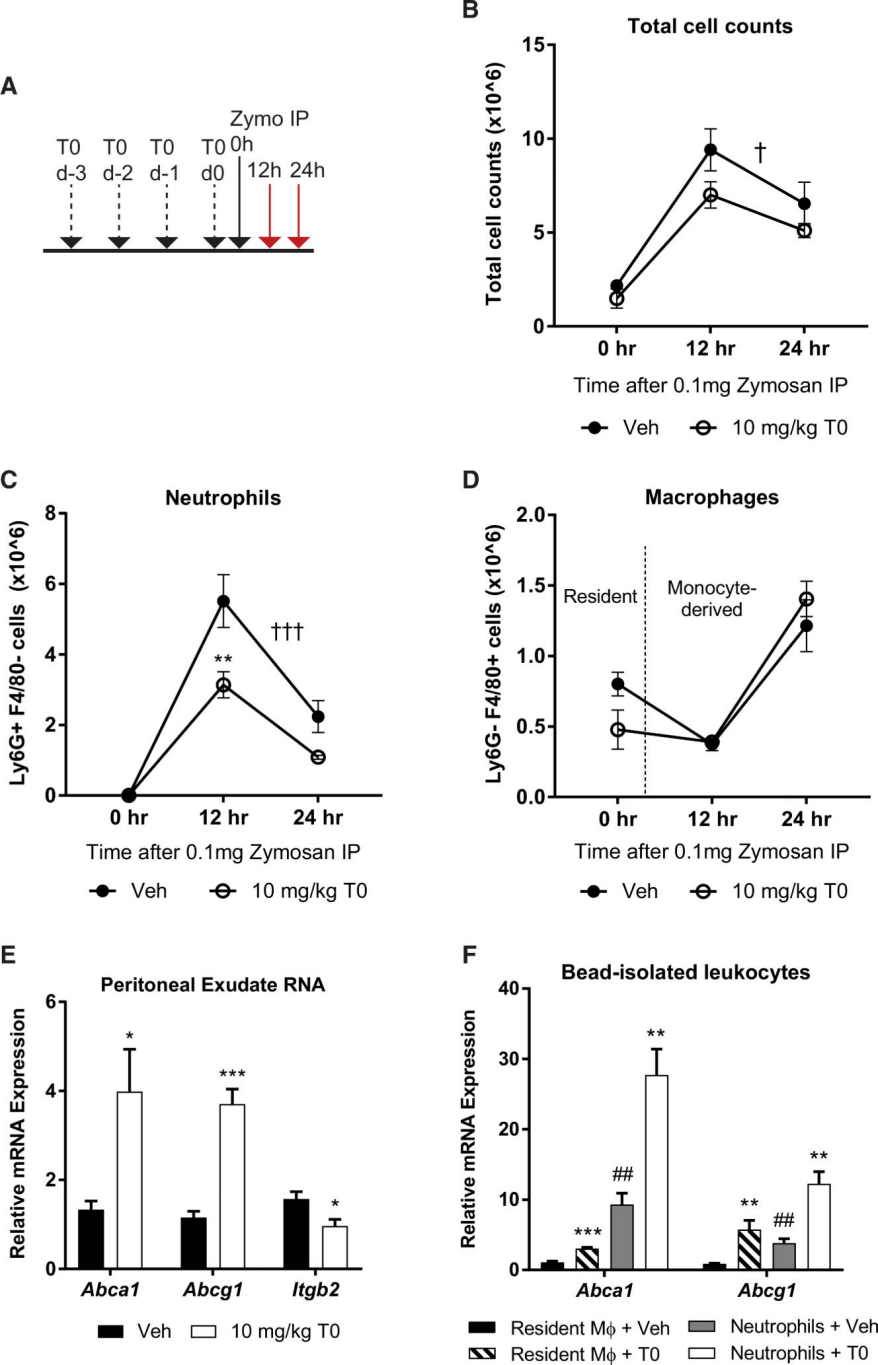
LXR Activation Suppresses Neutrophil Migration *In Vivo* Mice were treated with T0 or vehicle before induction of zymosan peritonitis. (A) Dosing schedule for treatments and harvest of peritoneal exudates. (B–D) Total peritoneal exudate cell (B), neutrophil (C), and macrophage (D) counts at 0, 12, or 24 hr after zymosan injection. Cell counts were determined by flow cytometry. (E) Peritoneal exudate cell mRNA expression at 4 hr after zymosan injection was measured by qPCR. (F) Peritoneal exudate leukocyte subsets were isolated using anti-Ly6G- or anti-F4/80-conjugated magnetic beads at 4 hr after zymosan injection and mRNA expression was measured by qPCR. Mean ± SEM is plotted; n = 4–5 biological replicates. Significance was determined by two-way ANOVA with Sidak’s post hoc test (B–D) or by Student’s t test with Benjamini-Hochberg multiple testing correction (E and F). *p < 0.05, **p < 0.01, and ***p < 0.001 for individual time point; †p < 0.05, ††p < 0.01, and †††p < 0.001 for treatment effect by 2-way ANOVA; #p < 0.05, ##p < 0.01, and ###p < 0.001 for leukocyte subset effect. Data are representative of two independent experiments. See also Figure S4.

The repression of genes associated with leukocyte cell-cell adhesion and granulocyte chemotaxis in inflammatory macrophages led us to consider whether LXR activation would block neutrophil recruitment during sterile inflammation. We used the yeast cell wall component zymosan A to elicit sterile peritonitis, which is characterized by infiltration of neutrophils in the onset phase 4–24 hr after zymosan injection, followed by resolution over ~3 days (Newson et al., 2014). Mice were treated for 3 days with 10 mg/kg T0 by daily oral gavage and given a final dose 2 hr before intraperitoneal injection of 0.1 mg zymosan (Figure 6A). Peritoneal exudates were collected 0, 12, and 24 hr after zymosan injection, and peritoneal exudate cells were identified as neutrophils (Ly6G+) or macrophages (F4/ 80+) by flow cytometry.

LXR agonist treatment leads to an overall decrease in peritoneal exudate cell counts (p < 0.05 by two-way ANOVA; Figure 6B). This effect is largely driven by a 44% decrease in neutrophil recruitment during inflammation onset (Figure 6C). Recruitment of monocyte-derived macrophages starts between 12 and 24 hr after zymosan injection, at which point resident macrophages are no longer recovered (Bannenberg et al., 2005), and is unchanged in T0-treated mice when compared to vehicle-treated controls (Figure 6D). Ly6G- F4/80- peritoneal exudate cell counts are also unchanged (Figure S4A). Blood neutrophil counts are unchanged after 3 days of T0 treatment, suggesting that the effect of T0 on exudate neutrophil counts is a consequence of defective neutrophil recruitment (Figure S4B). Exudate protein content reflects leakage of plasma proteins into the peritoneum (Bannenberg et al., 2005) and is unchanged after T0 treatment (Figure S4C), suggesting a cellintrinsic mechanism for the defect in neutrophil recruitment.

The specific defect in neutrophil migration suggests a potential role for LXR in regulating genes involved in lipid metabolism or cell adhesion in these cells. Thus, we assayed mRNA from neutrophil-rich early peritoneal exudates (4 hr after zymosan administration) and found that the LXR targets *Abca1* and *Abcg1* are induced, while the adhesion molecule *Itgb2* is repressed (Figure 6E). Interestingly, we found that *Abca1* and *Abcg1* are highly expressed and induced by T0 in isolated Ly6G+ exudate neutrophils (Figure 6F), suggesting that T0 has a cell-intrinsic effect on neutrophil cholesterol metabolism. Expression of *Cox2* and *Il1b*, on the other hand, is unchanged in peritoneal exudate mRNA, potentially due to the high level of inflammatory gene expression elicited by zymosan exposure, which exceeds the level of induction of these genes at which they are sensitive to LXR repression *in vitro*, similar to the effects of high levels of LPS (Figures S4D and S4E). These results suggest that LXR may suppress neutrophil migration through both metabolic and anti-inflammatory activities.

## DISCUSSION

LXR agonists suppress inflammation, which has stimulated widespread interest in their development as therapeutics for diseases such as dermatitis, rheumatoid arthritis, and atherosclerosis (Joseph et al., 2003; Kappus et al., 2014; Li et al., 2010). This activity has been linked to either LXR transrepression or cholesterol efflux, but recent studies have challenged the role of transrepression by LXR in control of inflammation (Ito et al., 2015). We confirmed a role for cholesterol efflux in LXR repression but uncovered a *cis*-repressive activity of LXR acting at inflammatory gene enhancers that plays a major role in LXR repression. We further established that this activity targets genes associated with several pro-atherogenic leukocyte functions, including neutrophil migration, and found that LXR agonist treatment blocks neutrophil recruitment during sterile inflammation.

The direct repressive effect of LXR agonists has been attributed to an NCoR-dependent repressive function of SUMOylated LXR acting generally at the stimulus-dependent transcription factor AP-1 (Ghisletti et al., 2009). This model suggests that during LXR repression, LXR binds indirectly to corepressor complexes around AP-1 response elements without a defined role for the DNA-binding domain of LXR. In contrast, we find that the direct repressive activity of LXR is associated with binding in *cis* to LXR response elements and does not appear to require AP-1 activity, indicating that LXR repression is targeted by genome-encoded regulatory interactions to certain inflammatory genes where LXR binds directly. This proposed mechanism of repression is similar to the *cis*-repressive activity of the glucocorticoid receptor (Uhlenhaut et al., 2013). By comparison, cholesterol efflux-dependent anti-inflammatory functions of LXR, which interfere with TLR signaling, have been shown to broadly interfere with TLR signaling and inflammatory gene activation (Ito et al., 2015; Westerterp et al., 2013).

The combined activities of LXR repression attenuate the expression of neutrophil cell adhesion and migration genes, and we observed that LXR activation limits neutrophil recruitment during sterile peritonitis. Our data suggest that the *cis*-repressive function of LXR, by suppressing integrin gene expression, may play a role in this activity. In addition, we found that neutrophil expression of cholesterol efflux transporters is robust and strongly stimulated by T0. A role for cholesterol efflux in limiting inflammatory migration of these cells is consistent with previous reports that ABCA1 activity is associated with decreased migration in macrophages (Zhu et al., 2012). Thus, it is likely that both *cis*-repression and cholesterol efflux contribute to the efficacy of T0 in the suppression of neutrophilic inflammation.

In atherosclerosis, LXR agonists are protective even in the absence of cholesterol efflux transporters, highlighting the importance of the dual functions of LXR in metabolism and inflammatory gene control (Kappus et al., 2014). The repression of neutrophil migration genes by LXR agonists may be particularly important in this context, as mice with cholesterol efflux transporter deficiency in myeloid cells have prominent neutrophil accumulation and neutrophil extracellular trap formation in lesions (Westerterp et al., 2018). Although LXR agonists have hepatotoxic effects, targeting metabolic and inflammatory functions of neutrophils or macrophages by activating LXR in these cells specifically remains a promising therapeutic strategy for the treatment of atherosclerosis.

## STAR⋆METHODS

### CONTACT FOR REAGENT AND RESOURCE SHARING

Further information and requests for resources and reagents should be directed to and will be fulfilled by Alan R. Tall (art1@columbia.edu).

### EXPERIMENTAL MODEL AND SUBJECT DETAILS

#### Animals

Wild-type C57BL/6J mice were obtained from The Jackson Laboratory (stock #000664). LXR KO (*Nr1h3*^*–/–*^ *Nr1h2*^*–/*–^) mice were generated as described previously (Alberti et al., 2001) and were backcrossed into the C57BL/6J background for at least 10 generations. *Myd88*^–/–^ mice were obtained from The Jackson Laboratory (stock #009088) and were backcrossed into the C57BL/6J background for at least 10 generations. *LysMCre Abca1*^*fl/fl*^*Abcg1*^*fl/fl*^ and littermate control *Abca1*^*fl/fl*^*Abcg1*^*fl/fl*^ mice were generated as described previously (Westerterp et al., 2013). *Lpcat3*^*fl/fl*^ were generated as described previously (Kabir et al., 2016) and crossed with *LysMCre* mice from The Jackson Laboratory (stock #004781) to generate mice with myeloid *Lpcat3* deficiency and littermate controls.

All mice were housed at Columbia University Medical Center according to animal welfare guidelines. Animals were kept under specific pathogen-free conditions with ad *libitum* access to both food and water. Mice were fed irradiated chow diet (Purina Mills diet 5053). Housing temperatures were kept within a range of 71–73°F (21.7–22.8°C). Water and cages were autoclaved and cages were changed once weekly. The health status of the mice was monitored using a dirty bedding sentinel program and no health status issues or changes in immune status were identified. Mice were not used for any procedures prior to bone marrow isolation or peritonitis experiments. Female mice aged 8–12 weeks (weight 18–25 g) were used for all experiments. For *in vivo* peritonitis experiments, age-matched mice were randomly assigned to treatment or control groups. No inclusion or exclusion criteria were used. All protocols were approved by the Institutional Animal Care and Use Committee of Columbia University.

#### Primary Cell Culture

For generation of bone marrow-derived macrophages, female mice aged 8–10 weeks were euthanized in accordance with American Veterinary Association Panel on Euthanasia regulations and bone marrow was isolated from femurs and tibias. Bone marrow cells were differentiated into macrophages by culture in DMEM 10% FBS, 1% pen-strep supplemented with 20% L-cell conditioned medium in tissue culture treated plates in an incubator set at 37°C and 5% CO_2_. After 7 days, macrophages were fully differentiated and subjected to a one-day serum deprivation in DMEM, 1% pen-strep supplemented with 4% L-cell conditioned medium to normalize exposure to serum-derived lipoproteins before treatment with LXR agonist and inflammatory agents as described in Method Details.

### METHOD DETAILS

#### Zymosan Peritonitis

Female mice aged 10–12 weeks were randomly assigned to vehicle or LXR agonist treatment groups. Mice were pre-treated with 10 mg/kg T0901317 (Selleckchem) prepared in 0.9% carboxymethylcellulose solution, or vehicle alone, for 3 days by daily oral gavage. Twenty-four hours after the 3rd dose, mice were treated once with 10 mg/kg T0901317 prepared as above, or vehicle alone, by oral gavage 2 hours before intraperitoneal injection of 0.1 mg zymosan (Sigma) in 0.5 mL sterile PBS. At 4, 12, or 24 hours after zymosan treatment, or without zymosan injection, mice were euthanized in accordance with American Veterinary Association Panel on Euthanasia regulations. Peritoneal exudates were harvested and cells were stained with anti-F4/80 clone BM8 (eBioscience) and anti-Ly6G clone 1A8 (BioLegend) for analysis of cell counts by flow cytometry or isolated with anti-F4/80 or anti-Ly6G microbeads (Miltenyi) for RNA analysis. For blood neutrophil counts, blood was collected by cardiac puncture and treated with RBC lysis buffer (Biolegend). Blood cells were stained with anti-CD115 clone AFS98 (Thermo) and anti-Gr1 clone RB6–8C5 (BD Biosciences) for analysis of cell counts by flow cytometry. Exudate protein content was measured by bicinchoninic acid (BCA) assay (Pierce). For peritonitis experiments, data is representative of two independent experiments and 4–6 mice were used for each condition, as indicated in the figure legends.

#### BMDM Treatment and Stimulation

For LXR agonist treatment and inflammatory stimulation of macrophages, BMDM were treated with LXR agonist T0901317 (Selleckchem) at a concentration of 500 nM or DMSO vehicle alone at a 1:10,000 dilution in serum-free medium with 4% L-cell conditioned medium for 3 hours. After this treatment period, macrophages were harvested directly for transposase-accessible DNA isolation from unstimulated cells or stimulated with 10 ng/mL LPS (Cell Signaling) added directly to agonist-containing medium for 2 hours before transposase-accessible DNA or RNA isolation. For knockdown experiments, macrophages were differentiated as described above and treated on day 7 with 100 nM SMARTpool siRNA (Dharmacon) against *Scd2* or control non-targeting siRNA complexed with Lipofectamine RNAiMAX (Thermo) in OptiMEM medium (Thermo) for 24 hours. After this period, Optimem was aspirated and replaced with DMEM, 1% pen-strep supplemented with 4% L-cell supernatant for one additional day. On day 9, macrophages were treated with LXR agonist T0901317 at 500 nM for 3 hours and stimulated with 10 ng/mL LPS for 2 hours. For MAPK inhibitor experiments, macrophages were treated with 10 μM PD0325901 (Sigma) and 1 μM BIRB0796 (AXON Medchem) as described (Tong et al., 2016) starting at the same time as treatment with T0901317 at 500 nM for 3 hours before stimulation with 10 ng/mL LPS for 2 hours. For MERTK antibody neutralization experiments, anti-Mer blocking antibody AF591 (R&D Systems) was added 3 hours prior to treatment with T0901317 at 500 nM for 3 hours and stimulated with 10 ng/mL LPS for 2 hours. For each BMDM stimulation experiment, data is representative of two independent experiments and 3–4 independently differentiated macrophage cultures were used for each condition, as indicated in the figure legends.

#### Gene Expression Analysis

Macrophages were washed twice with cold PBS and lysed in RNA lysis buffer (QIAGEN or Zymo Research). RNA was isolated using RNeasy kits (QIAGEN) or RNA MiniPrep kits (Zymo Research). cDNA was prepared using first strand synthesis kits (Thermo) and qPCR was performed on an ABI StepOnePlus machine with SYBR reagents (Thermo). The following primers were used for qPCR analyses: Cox2-F: AACCGCATTGCCTCTGAAT; Cox2-R: CATGTTCCAGGAGGATGGAG (Nasser et al., 2012); *Il1b*-F: GCAACTG TTCCTGAACTCAACT; *Il1b*-R: ATCTTTTGGGGTCCGTCAACT (Huang et al., 2011); *Abca1*-F: CAGCTTCCATCCTCCTTGTC; *Abca1*-R: CCACATCCACAACTGTCTGG (Murphy et al., 2013); Abcg1-F: GTACCATGACATCGCTGGTG; *Abcg1*-R: AGCCGTA GATGGACAGGATG (Murphy et al., 2013); *Itgb2*-F: CCCAGGAATGCACCAAGTACA; *Itgb2*-R: CAGTGAAGTTCAGCTTCTGGCA

(generated for this paper).

#### ATAC-seq Experimental Preparation

Samples were prepared for ATAC-seq essentially as described previously (Buenrostro et al., 2013). Macrophages were washed twice in cold PBS, scraped in cold PBS, and counted using a hemocytometer. Based on this count, 50,000 cells were aliquoted and pelleted by centrifugation. Cell pellets were washed once with 50 μL cold PBS on ice before lysis in 50 μL hypotonic lysis buffer (10 mM Tris-HCl pH 7.5, 10 mM NaCl, 3 mM MgCl2, 0.1% IGEPAL CA-630) over the course of a 10 minute spin at 4°C. Pelleted nuclei were resuspended in 50 uL transposition reaction mix with 3 uL Nextera transposase per sample (Illumina). The reaction was stopped with 0.1% SDS and transposase-accessible DNA was isolated using AMPure XP beads (Beckman-Coulter). Accessible DNA was amplified by PCR for 5 cycles, assessed for yield by qPCR, and amplified for an additional 7 cycles. Libraries were sequenced on a NextSeq 500 (Illumina).

#### ATAC-seq Data Processing

ATAC data from each sample was aligned using Bowtie2 (Langmead and Salzberg, 2012) after adaptor trimming using cutadapt (Martin, 2011) and PCR duplicates were removed using samtools (Li et al., 2009). Quadruplicate samples for each condition were used for peak calling by MACS2 (Zhang et al., 2008) with the parameters -q 0.001–nomodel–shift 88–extsize 177 to set an FDR threshold of 0.1% and account for average insert size. Coverage tracks were created using deeptools (Ramírez et al., 2014) using reads per genomic content (RPGC) normalization and visualized in Integrative Genomics Viewer (Thorvaldsdóttir et al., 2013). To limit ATAC peak identification to transposase-accessible enhancers and exclude other accessible loci, ATAC peaks were filtered according to correspondence with H3K27ac- or H3K4me2-marked macrophage enhancers in resting or stimulated primary macrophages identified previously (Oishi et al., 2017). Enhancer overlap, overlap between conditions, and nearest gene annotation were performed using bedtools (Quinlan and Hall, 2010). Gene Ontology analysis of nearest genes for each enhancer set was performed using the PANTHER database (Thomas et al., 2003) and sequence motif analysis was performed using HOMER (Heinz et al., 2010). Motif analysis for T0-closed and LPS-opened enhancers was performed using the full set of unstimulated macrophage enhancers as sequence background, while motif analysis for T0-closed-in-LPS enhancers was performed using the full set of LPS-stimulated enhancers as sequence background. Enhancers were aligned to LXR ChIP-seq data from Oishi et al., 2017 using deeptools (Ramírez et al., 2014), and enhancer-TSS distances were computed using custom scripts deposited at https://github.com/dgt2109/bio-script.

#### RNA-seq

For RNA-seq, macrophages were washed twice with cold PBS and lysed in TRIzol reagent (Thermo). RNA was isolated from the aqueous phase using RNeasy kits (QIAGEN). RNA with RIN > 8 was subjected to poly-dT pulldown using magnetic beads (NEB) before preparation for RNA-seq using RNA Ultra kits (NEB). Libraries were sequenced on a NextSeq 500 (Illumina) and reads were aligned to the mm10 transcriptome using HISAT2 (Kim et al., 2015) after adaptor trimming using cutadapt (Martin, 2011). Reads counts per gene for RefSeq genes were computed using featureCounts (Liao et al., 2014). Counts were normalized to reads per kilobase per million (RPKM) and processed for pairwise differential expression analysis of selected conditions using DESeq2 (Love et al., 2014) with a False Discovery Rate (FDR)-adjusted p value cutoff of 0.05. Gene Ontology analysis was performed using the PANTHER database (Thomas et al., 2003).

#### Measurement of Phosphatidylcholine Subspecies

Phosphatidylcholine subspecies of BMDM were measured using infusion-based high-resolution mass spectrometry as described previously (Li et al., 2012) using a Triple TOF 5600 (AB-Sciex). Lipids were extracted using the Bligh/Dyer method (Bligh and Dyer, 1959) after addition of internal standards and data were acquired on a Triple TOF 5600 operated in TOF mode at a resolution of 35,000, electrospray source voltage of 5500 v on the Turbo B spray interface, declustering potential of 100 V, scanning from 100 to 1200 Da. Samples were infused at ~20 μL/min in a solution of 4:2:1 isopropanol:methanol:chloroform with 10 mM ammonium acetate with a Reliance autosampler (Sparck) operating in pressurized vessel mode. Quantitation was performed using MultiQuant (AB-Sciex). A window of ± 5 mDa was used to identify PC species. Curves were calculated using 1/X weighting and were applied uniformly.

### QUANTIFICATION AND STATISTICAL ANALYSIS

All data are presented as mean ± SEM. In BMDM experiments, sample size (*n*) represents the number of individually differentiated primary macrophage cultures in each experiment. In peritonitis experiments, sample size (*n*) represents the number of individual mice in each experiment. The statistical parameters (*n*, mean, SEM, and statistical tests used) can be found within the figure legends and figures. For comparisons of 2 datasets, the Student’s t test with Benjamini-Hochberg multiple testing correction was used to determine significance. For comparison of 3 or 4 datasets, one-way ANOVA with Tukey’s post hoc test was used, except in the case of time-course data where two-way ANOVA with Sidak’s post hoc test was used to determine significance. For RNA-seq, gene expression differences were evaluated by Wald test after linear model fitting using DESeq2 and genes significant at 5% FDR were considered to be differentially expressed. ATAC-seq peaks were identified using Model-Based Analysis of ChIP-seq 2 (MACS2) software. Enhancer-gene distance distributions were compared using Kruskal-Wallis nonparametric one-way ANOVA with Dunn post hoc test. The criterion for significance was set at p < 0.05. No inclusion or exclusion criteria were used. No statistical method was used to determine whether the data met assumptions of the statistical approach. Statistical analyses were performed using GraphPad Prism version 7.0.3 or R software with the indicated packages for sequencing data.

### DATA AND SOFTWARE AVAILABILITY

The NCBI GEO accession numbers for high throughput sequencing data reported in this paper are GEO: GSE110002, GSE109997, and GSE109998. Custom scripts for enhancer-promoter distance calculation are deposited at https://github.com/dgt2109/bio-script.

## Supplementary Material

1

2

## Figures and Tables

**KEY RESOURCES TABLE:** 

REAGENT or RESOURCE	SOURCE	IDENTIFIER
Antibodies		
F4/80-Pacific Blue	Thermo	Cat# MF48028; RRID:AB_10373419
Ly6G-FITC	BioLegend	Cat# 127605; RRID:AB_1236488
Mer neutralizing antibody	R&D Systems	Cat# AF591; RRID:AB_2098565
Normal goat IgG control	R&D Systems	Cat# AB-108-C; RRID:AB_354267
Chemicals, Peptides, and Recombinant Proteins		
T0901317	Selleckchem	Cat# S7076
Lipopolysaccharide	Cell Signaling	Cat# 14011
Lipofectamine RNAiMAX	Thermo	Cat# 13778075
Scd2 SMARTpool siRNA	Dharmacon/GE	Cat# L-045507–01-0005
Non-targeting control SMARTpool siRNA	Dharmacon/GE	Cat# D-001810–10-05
PD0325901	Sigma	Cat# PZ0162
BIRB0796	AXON Medchem	Cat# 1358
Critical Commercial Assays		
Nextera DNA library prep kit	Illumina	Cat# FC-121–1030
NEBNext RNA Ultra library prep kit	NEB	Cat# E7530S
F4/80 microbeads, ultrapure, mouse	Miltenyi	Cat# 130–110-443
Ly6G microbeads, mouse	Miltenyi	Cat# 130–092-332
BCA protein assay kit	Pierce	Cat# 23225
Deposited Data		
LXR agonist ATAC-seq	This paper	GEO: GSE109998
LXR agonist RNA-seq	This paper	GEO: GSE109997
LXR ChIP-seq	Oishi et al., 2017	GEO: GSE79423
H3K27ac ChIP-seq	Oishi et al., 2017	GEO: GSE79423
H3K4me2 ChIP-seq	Oishi et al., 2017	GEO: GSE79423
Experimental Models: Organisms/Strains		
mouse: *Nr1h3*^−/−^ *Nr1h2*^*−/−*^; B6.129S1.129X1-*Nr1h2*^*tm1.1Gstr*^ *Nr1h2*^*tm1.1Gstr*^	Alberti et al., 2001	MGI Cat# 2653351, RRID:MGI:2653351
mouse: *Myd88*^−/−^; B6.129P2(SJL)-*Myd88*^*tm1.1Defr*^/J	The Jackson laboratory	JAX Cat# 009088; RRID:MGI:4430210
mouse: *Abca1*^*fl/fl*^*Abcg1*^*fl/fl*^; B6.Cg-*Abca1*^*tm1Jp*^ *Abcg1*^*tm1Tall*^/J	Westerterp et al., 2013	JAX Cat# 021067; RRID:IMSR_JAX:021067
Mouse: *Lpcat3*^*fl/fl*^; B6.Cg-*Lpcat3*^*tm1*.1*Igl*^/N	Kabir et al., 2016	MGI Cat# 6158452; RRID: N/A
mouse: *LysMCre*;B6.129P2-*Lyz2*^*tm1(cre)Ifo*^/J	The Jackson laboratory	JAX Cat# 004781; RRID:IMSR_JAX:004781
Oligonucleotides		
Mouse Cox2 forward primer: AACCGCATTGCCTCTGAAT	Nasser et al., 2012	N/A
Mouse Cox2 reverse primer: CATGTTCCAGGAGGATGGAG	Nasser et al., 2012	N/A
Mouse Il1b forward primer: GCAACTGTTCCTGAACTCAACT	Huang et al., 2011	N/A
Mouse Il1b reverse primer: ATCTTTTGGGGTCCGTCAACT	Huang et al., 2011	N/A
Mouse Abca1 forward primer: CAGCTTCCATCCTCCTTGTC	Murphy et al., 2013	N/A
Mouse Abca1 reverse primer: CCACATCCACAACTGTCTGG	Murphy et al., 2013	N/A
Mouse Abcg1 forward primer: GTACCATGACATCGCTGGTG	Murphy et al., 2013	N/A
Mouse Abcg1 reverse primer: AGCCGTAGATGGACAGGATG	Murphy et al., 2013	N/A
Mouse Itgb2 forward primer: CCCAGGAATGCACCAAGTACA	This paper	N/A
Mouse Itgb2 reverse primer: CAGTGAAGTTCAGCTTCTGGCA	This paper	N/A
Software and Algorithms		
Graphpad Prism v7.0.3	GraphPad Software	https://www.graphpad.com/scientificsoftware/prism/
cutadapt v1.14	Martin, 2011	https://cutadapt.readthedocs.io/en/stable/guide.html
samtools v1.5	Li et al., 2009	http://samtools.sourceforge.net/
MACS2 v2.1.1	Zhang et al., 2008	https://github.com/taoliu/MACS
Deeptools v2.5.4	RamÍrez et al., 2014	https://deeptools.readthedocs.io/en/develop/
Integrative Genomics Viewer v2.4.14	Thorvaldsdóttir et al., 2013	http://software.broadinstitute.org/software/igv/
bedtools v2.25.0	Quinlan and Hall, 2010	https://bedtools.readthedocs.io/en/latest/
PANTHER GO	Thomas et al., 2003	http://www.geneontology.org/page/goenrichment-analysis
HOMER v4.9.1	Heinz et al., 2010	http://homer.ucsd.edu/homer/
HISAT2 v2.1.0	Kim et al., 2015	https://ccb.jhu.edu/software/hisat2/index.shtml
featureCounts v1.5.0r	Liao et al., 2014	http://subread.sourceforge.net/
DESeq2 v1.18.1	Love et al., 2014	https://bioconductor.org/packages/release/bioc/html/DESeq2.html
FACS DiVa	BD Biosciences	http://www.bdbiosciences.com/us/instruments/research/software/flowcytometry-acquisition/bd-facsdivasoftware/m/111112/overview
FlowJo	FlowJo	https://www.flowjo.com/solutions/flowjo
MultiQuant	AB-Sciex	https://sciex.com/products/software/multiquant-software
promoter-enhancer_dist.sh	This paper	https://github.com/dgt2109/bio-script

## Highlights

- Cholesterol efflux and direct repression mediate LXR’s anti-inflammatory effects
- LXR *cis*-repression involves direct binding of LXR to inflammatory gene enhancers
- LXR agonist treatment suppresses neutrophil recruitment during inflammation
- Targets of LXR repression are involved in neutrophil migration
