# Supplementary material for: LXR Suppresses Inflammatory Gene Expression and Neutrophil Migration through cis-Repression and Cholesterol Efflux
Source: Cell Rep. Author manuscript; Available in PMC 2019 Apr 3. (PMC6446575; doi:10.1016/j.celrep.2018.11.100)
Supplement: 1 [file NIHMS1517595-supplement-1.pdf]

Cell Reports, Volume *25*

Supplemental Information

LXR Suppresses Inflammatory Gene Expression and Neutrophil Migration through *cis*-Repression and Cholesterol Efflux

David G. Thomas, Amanda C. Doran, Panagiotis Fotakis, Marit Westerterp, Per Antonson, Hui Jiang, Xian-Cheng Jiang, Jan-Åke Gustafsson, Ira Tabas, and Alan R. Tall

Supplemental Figures

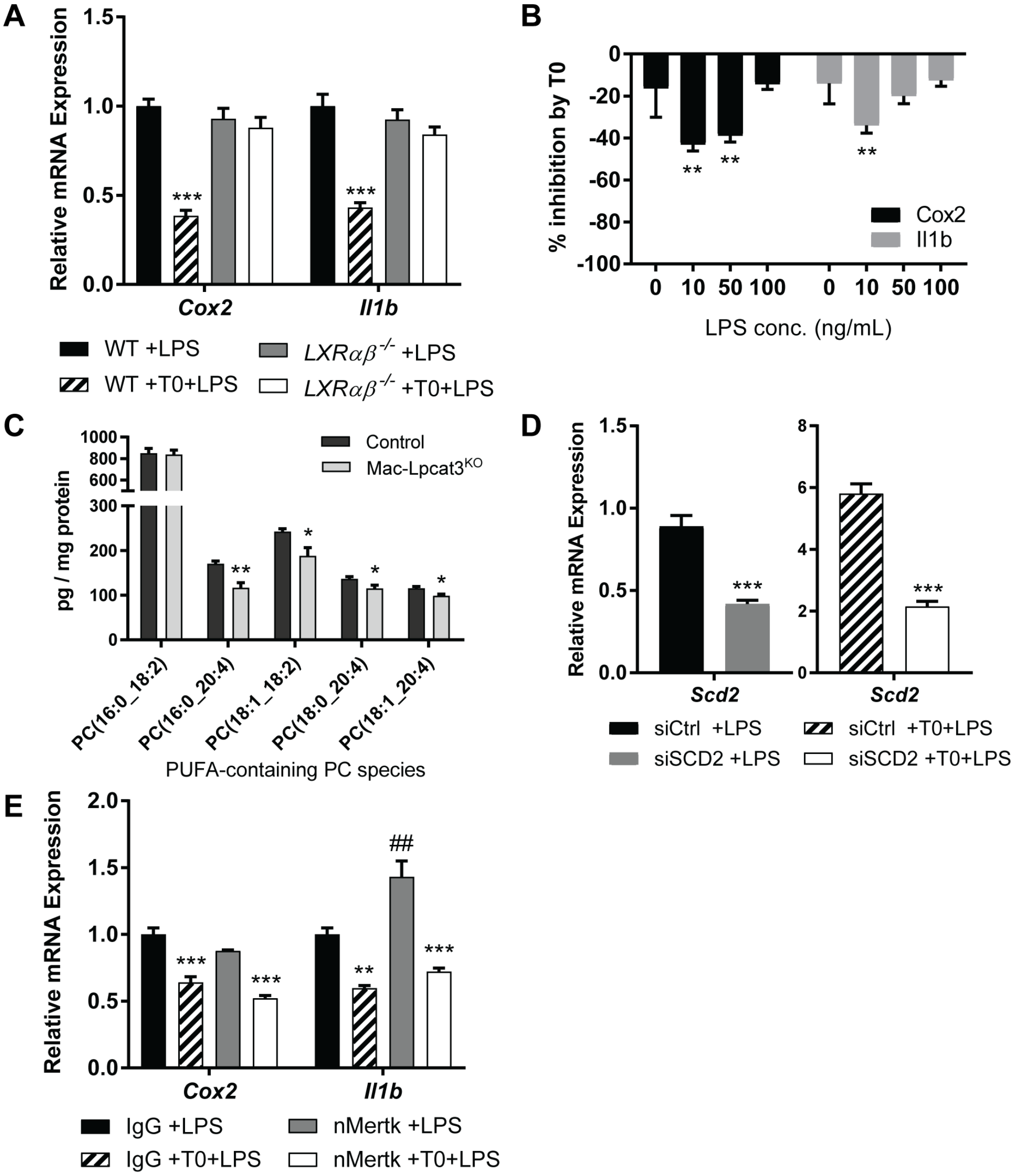

Figure S1; Related to Figure 1. Specificity and validation of *in vitro* models of LXR repression. *(A)* WT or *Nr1h3*$^{-/-}$ *Nr1h2*$^{-/-}$ (*LXRαβ*$^{-/-}$) BMDM were treated for 3 hours with 500 nM T0 before stimulation with 10 ng/mL LPS for 2 hours. (*B)* WT BMDM were treated for 3 hours with 500 nM T0 before stimulation with 0, 10, 50, or 100 ng/mL LPS as indicated. *(C)* Phosphatidylcholine (PC) species of WT and *LysMCre Lpcat3*$^{fl/fl}$ (Mac-Lpcat3^{KO}) BMDM were measured by infusion-based high-resolution mass spectroscopy. Relative levels of PUFA-containing PC are shown. *(D)* BMDM were treated for 24 hours with an siRNA Smartpool targeted against SCD2 (siSCD2) or with a nontargeting control Smartpool (siCtrl) and then rested for 24 hours. *Scd2* mRNA expression was determined at the time of experiment 48 hours after transfection. *(E)* BMDM were pretreated with MERTK neutralizing antibody (nMERTK) or control goat IgG for 3 hours at a concentration of 20 µg/mL and then treated as in (A). mRNA expression was evaluated by qPCR. Mean +/- SEM is plotted. n = 4 biological replicates (A-B, D-E) or 5-6 biological replicates (C). Significance was determined by one-way ANOVA with Tukey's post-hoc test (A, E), two-way ANOVA with Sidak's post-hoc test (B), or Student's t-test with Benjamini-Hochberg multiple testing correction (C, D). (*) $P < 0.05$, (**) $P < 0.01$, (***) $P < 0.001$ for T0 treatment vs control. (#) $P < 0.05$, (##) $P < 0.01$, (###) $P < 0.001$ for nMERTK effect vs IgG.

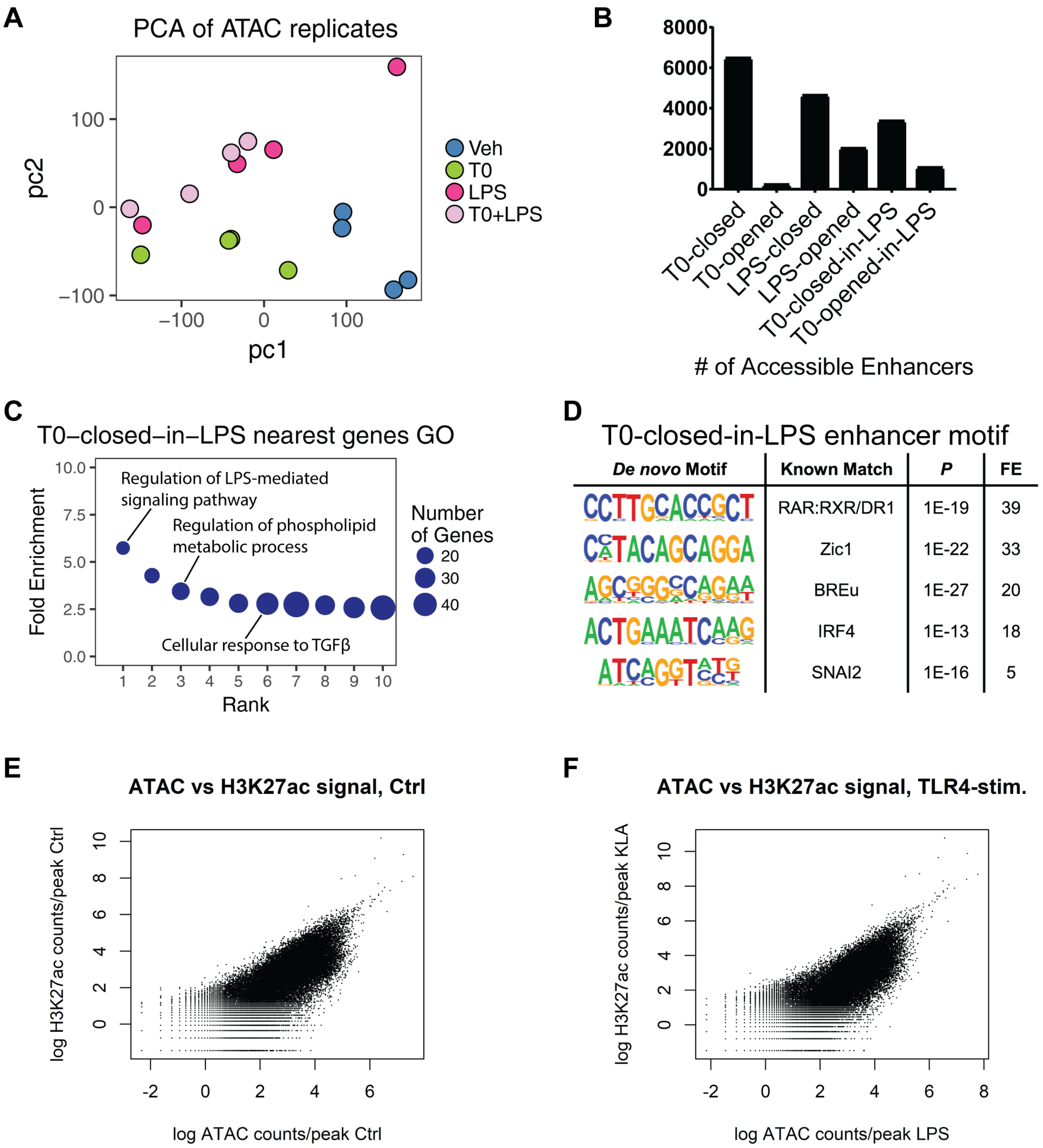

Figure S2; Related to Figure 2. Characterization of T0-related chromatin changes by ATAC-seq. *(A)* PCA analysis of ATAC signal over all macrophage enhancers by replicate. *(B)* Number of enhancers opened or closed by T0 and LPS treatments. *(C)* PANTHER GO categories enriched in genes nearest to T0-closed-in-LPS enhancers (Bonferroni-adjusted $P < 0.05$). *(D)* HOMER *de novo* motifs enriched in T0-closed-in-LPS enhancers ($P < 1 \times 10^{-12}$; top 5 motifs displayed). FE: fold enrichment. *(E-F)* Correlation of ATAC signal with H3K27ac signal from Oishi et al., 2017 over macrophage enhancers from untreated macrophages (E) or TLR4-stimulated (TLR4-stim.) macrophages (F). $n = 4$ biological replicates (ATAC-seq) or 1 biological replicate (H3K27ac ChIP-seq).

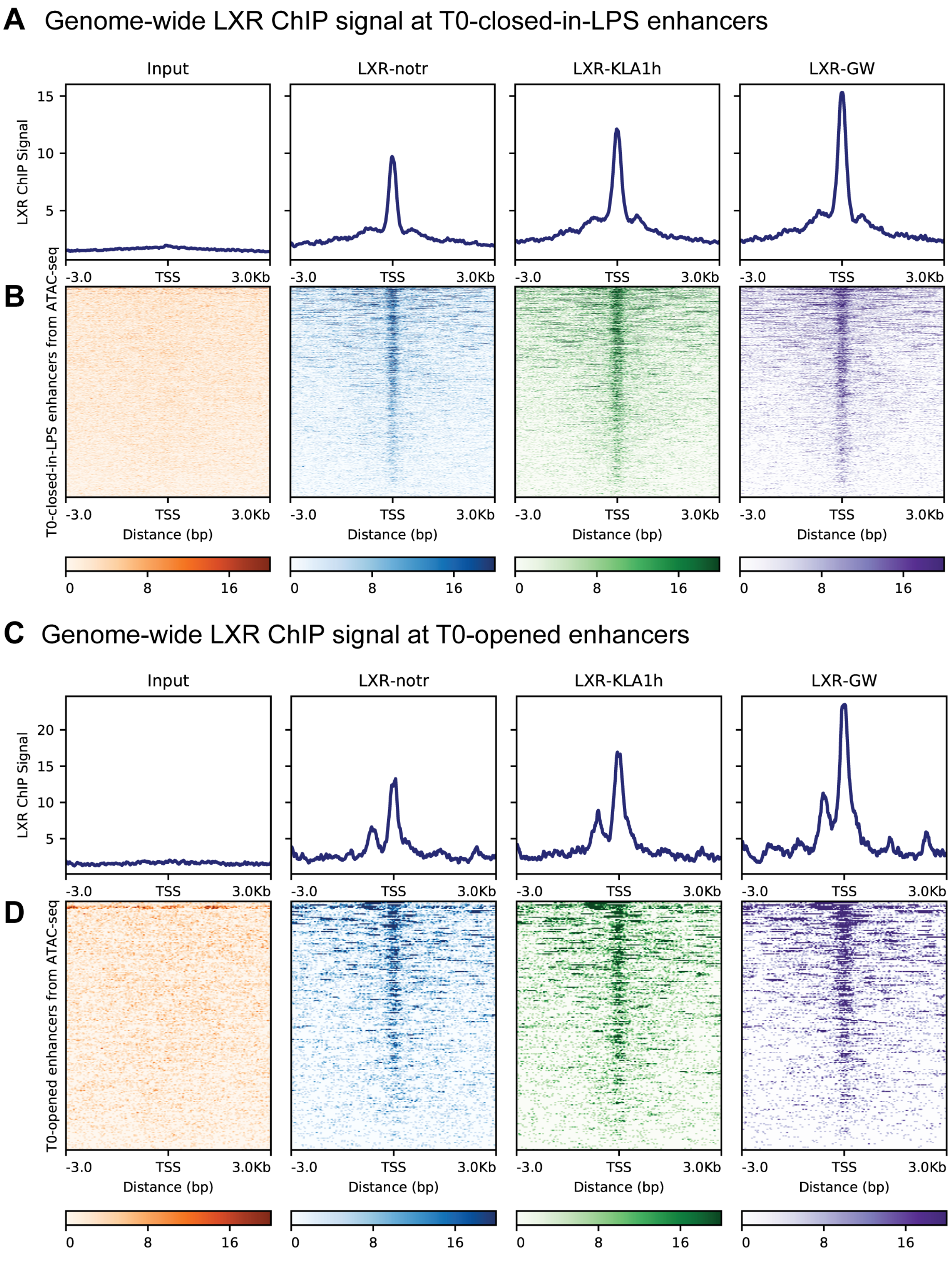

Figure S3; Related to Figure 3. LXR ChIP signal is present at T0-closed-in-LPS and T0-opened enhancers. *(A)* Histogram of LXR ChIP-seq signal from Oishi et al., 2017 centered on T0-closed-in-LPS enhancers. LXR-notr: chromatin from resting thioglycolate-elicited macrophages (TGEM) immunoprecipitated with anti-LXR antibody (notr: no treatment); LXR-KLA1h: chromatin from TGEM stimulated with TLR4 agonist KLA for 1 hour; LXR-GW: chromatin from TGEM treated with LXR agonist GW3965 for 24 hours. *(B)* Heatmap of LXR ChIP-seq signal as in (A) centered on T0-closed-in-LPS enhancers. *(C)* Histogram of LXR ChIP-seq signal as in (A) centered on T0-opened enhancers. *(D)* Heatmap of LXR ChIP-seq signal as in (A) centered on T0-opened enhancers. $n = 4$ biological replicates (ATAC-seq) or 1 biological replicate (LXR ChIP-seq).

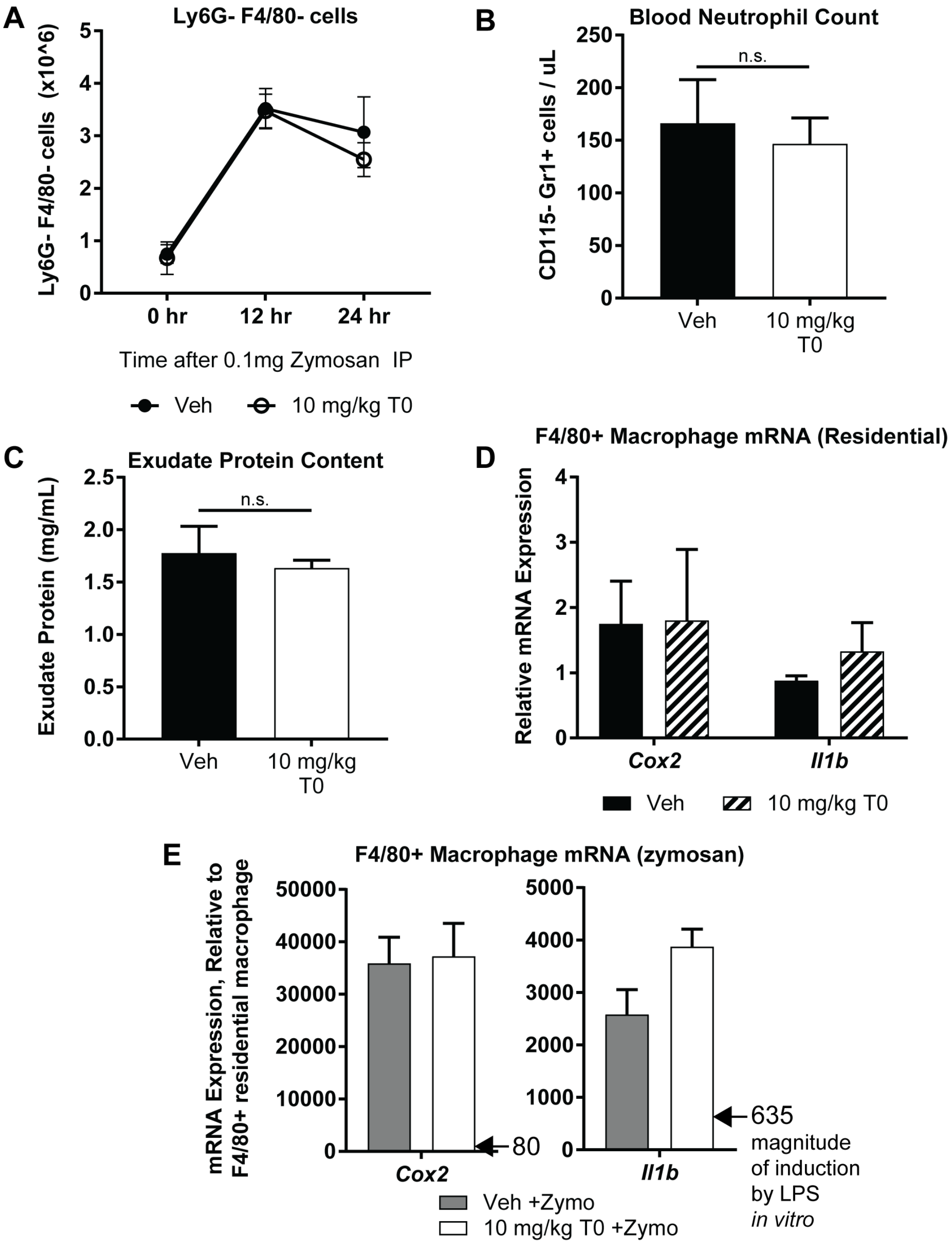

Figure S4; Related to Figure 6. Effect of T0 on parameters related to zymosan peritonitis severity. *(A)* Ly6G- F4/80- cell counts at 0, 12, or 24 hours after zymosan injection were determined by flow cytometry. *(B)* Circulating neutrophil counts with or without T0 treatment were determined by flow cytometry. *(C)* Level of exudate protein determined by BCA assay at 2h after zymosan injection. *(D)* Inflammatory gene expression in resident peritoneal macrophages with or without T0 treatment. *(E)* Inflammatory gene expression in inflammatory peritoneal macrophages 2h after zymosan injection. Arrows indicate magnitude of induction of each gene by 10 ng/mL LPS *in vitro*. n = 4-6 biological replicates (A-C, E) or 3 biological replicates (D). Significance was determined by two-way ANOVA with Sidak's post-hoc test (A) or by Student's t-test with Benjamini-Hochberg multiple testing correction (B-E). n.s.: not significant.

Supplemental Tables

Table S1; Related to Figure 2: ATAC-seq sequencing statistics. Quality control statistics for ATAC-seq data by condition are provided.

	**Veh**	**T0**	**LPS**	**T0+LPS**
**Replicates**	4	4	4	4
**Total Reads**	166,571,759	142,431,581	150,110,447	142,929,398
**Reads Mapped**	162,666,895	139,857,174	146,897,615	139,872,799
**Unique Reads Mapped**	68,804,852	67,716,584	64,099,577	59,297,287
**Fr. Reads in Peak (FRiP)**	0.143	0.094	0.122	0.109
**Mean Between-Replicate R^2**	0.972	0.974	0.973	0.983

Table S2; Related to Figure 2: Top 30 GO categories associated with genes nearest to T0-closed enhancers. Extended list of GO categories associated with genes nearest to T0-closed enhancers with detailed statistics, corresponding to Figure 2C.

GO biological process complete	# Genes	Fold Enrichment	Bonferroni -adjusted P
toll-like receptor signaling pathway (GO:0002224)	25	3.0	2.4E-02
negative regulation of peptidyl-tyrosine phosphorylation (GO:0050732)	27	2.8	2.3E-02
positive regulation of alpha-beta T cell activation (GO:0046635)	33	2.7	4.3E-03
transforming growth factor beta receptor signaling pathway (GO:0007179)	37	2.6	3.2E-03
endothelial cell differentiation (GO:0045446)	35	2.6	6.9E-03
regulation of phagocytosis (GO:0050764)	40	2.6	1.3E-03
negative regulation of response to wounding (GO:1903035)	36	2.5	1.5E-02
positive regulation of GTPase activity (GO:0043547)	95	2.4	2.8E-10
negative regulation of T cell activation (GO:0050868)	50	2.4	6.0E-04
modification of morphology or physiology of other organism involved in symbiotic interaction (GO:0051817)	37	2.4	2.9E-02
regulation of cell shape (GO:0008360)	63	2.3	1.7E-05
regulation of smooth muscle cell migration (GO:0014910)	36	2.3	4.2E-02
positive regulation of T cell differentiation (GO:0045582)	39	2.3	2.9E-02
positive regulation of smooth muscle cell proliferation (GO:0048661)	44	2.3	7.3E-03
cellular response to antibiotic (GO:0071236)	40	2.3	2.5E-02
regulation of Rho protein signal transduction (GO:0035023)	54	2.3	6.0E-04
regulation of leukocyte apoptotic process (GO:2000106)	40	2.3	3.2E-02
neural tube closure (GO:0001843)	44	2.2	1.5E-02
myeloid leukocyte differentiation (GO:0002573)	43	2.2	2.2E-02
T cell differentiation (GO:0030217)	62	2.2	2.0E-04
negative regulation of protein serine/threonine kinase activity (GO:0071901)	43	2.2	2.7E-02
regulation of gliogenesis (GO:0014013)	54	2.2	1.9E-03
establishment of cell polarity (GO:0030010)	46	2.2	1.8E-02
regulation of interleukin-6 production (GO:0032675)	52	2.2	4.9E-03
viral process (GO:0016032)	49	2.1	1.2E-02
receptor-mediated endocytosis (GO:0006898)	49	2.1	1.4E-02
regulation of plasma membrane bounded cell projection assembly (GO:0120032)	69	2.1	1.8E-04
regulation of I-kappaB kinase/NF-kappaB signaling (GO:0043122)	57	2.1	2.9E-03
wound healing (GO:0042060)	97	2.1	3.7E-07
positive regulation of leukocyte proliferation (GO:0070665)	55	2.1	6.0E-03

Table S3; Related to Figure 2: Full list of HOMER motifs for T0-closed enhancers. Extended list of HOMER categories associated with T0-closed enhancers with detailed statistics, corresponding to Figure 2D.

	***De novo* Motif**	**Known Match**	***P***	**FE**	**%Targets**	**%Background**
T0-closed	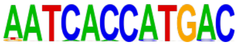	NR-half	1E-19	28	0.28	0.01
	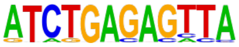	IRF4	1E-22	28	0.28	0.01
	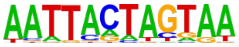	BCL6B	1E-27	28	0.28	0.01
	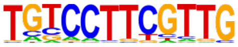	THRa/LXRE	1E-13	19	0.19	0.01
	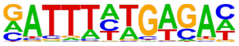	HOXA5	1E-16	19	0.19	0.01
	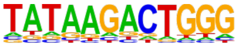	ZNF416	1E-13	19	0.19	0.01
	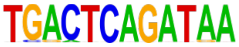	AP-1	1E-14	13	0.25	0.02
	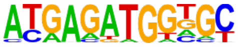	ZFP410	1E-12	11	0.22	0.02
	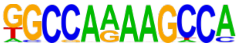	RXR/DR1	1E-14	9	0.36	0.04
	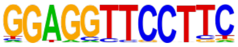	RELA	1E-14	6	0.51	0.09
	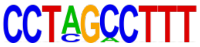	Nr4a1	1E-12	6	0.45	0.08
	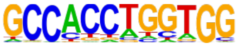	CTCFL	1E-28	2	3.51	1.53
	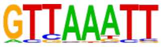	TCF1	1E-13	1.5	6.21	4.21
	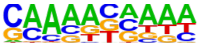	FOXL1	1E-13	1.3	10.46	7.85

Table S4; Related to Figure 2: Top 30 GO categories associated with genes nearest to LPS-opened enhancers. Extended list of GO categories associated with genes nearest to LPS-opened enhancers with detailed statistics, corresponding to Figure 2E.

GO biological process complete	# Genes	Fold Enrichment	Bonferroni-adjusted P
lipopolysaccharide-mediated signaling pathway (GO:0031663)	12	6	9.5E-03
regulation of p38MAPK cascade (GO:1900744)	14	6	3.6E-03
regulation of CD4-positive, alpha-beta T cell differentiation (GO:0043370)	15	5	1.5E-02
heart valve development (GO:0003170)	17	4	5.8E-03
negative regulation of smooth muscle cell proliferation (GO:0048662)	15	4	4.8E-02
positive regulation of T cell differentiation (GO:0045582)	26	4	2.8E-05
alpha-beta T cell activation (GO:0046631)	18	4	1.8E-02
positive regulation of alpha-beta T cell activation (GO:0046635)	17	4	4.0E-02
endothelial cell differentiation (GO:0045446)	19	4	1.2E-02
regulation of cell junction assembly (GO:1901888)	20	4	7.8E-03
T cell activation involved in immune response (GO:0002286)	18	4	3.2E-02
activation of innate immune response (GO:0002218)	19	4	1.7E-02
myeloid leukocyte differentiation (GO:0002573)	26	4	3.6E-04
positive regulation of histone modification (GO:0031058)	23	4	2.2E-03
cytokine production (GO:0001816)	36	4	2.1E-06
regulation of Rho protein signal transduction (GO:0035023)	30	3	1.3E-04
positive regulation of smooth muscle cell proliferation (GO:0048661)	24	3	4.1E-03
positive regulation of myeloid cell differentiation (GO:0045639)	21	3	2.9E-02
regulation of interferon-gamma production (GO:0032649)	23	3	1.0E-02
positive regulation of cytokine secretion (GO:0050715)	30	3	3.5E-04
response to transforming growth factor beta (GO:0071559)	27	3	1.8E-03
regulation of leukocyte apoptotic process (GO:2000106)	21	3	4.7E-02
protein dephosphorylation (GO:0006470)	35	3	4.5E-05
T cell differentiation (GO:0030217)	33	3	1.5E-04
cellular response to tumor necrosis factor (GO:0071356)	24	3	1.4E-02
negative regulation of cell-cell adhesion (GO:0022408)	38	3	1.6E-05
positive regulation of autophagy (GO:0010508)	22	3	4.0E-02
dendrite development (GO:0016358)	25	3	1.6E-02
negative regulation of protein secretion (GO:0050709)	28	3	4.3E-03
MAPK cascade (GO:0000165)	31	3	1.1E-03

Table S5; Related to Figure 2: Full list of HOMER motifs for LPS-opened enhancers. Extended list of HOMER categories associated with LPS-opened enhancers with detailed statistics, corresponding to Figure 2F.

	***De novo* Motif**	**Known Match**	***P***	**FE**	**%Targets**	**%Background**
LPS-opened	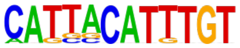	SpiB/ETS	1E-13	21	0.64	0.03
	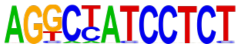	ATF2/Jun	1E-13	15	0.74	0.05
	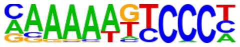	NFkB-p65 (1)	1E-24	4	3.76	0.86
	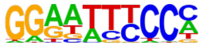	NFkB-p65 (2)	1E-162	4	25.25	6.26
	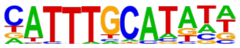	POU2F2/POU	1E-30	3	7.57	2.59
	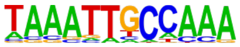	CEBPa	1E-12	3	3.02	1.05
	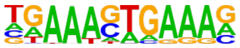	IRF1	1E-19	3	5.79	2.19
	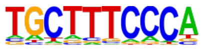	Rbpj1	1E-19	2	11.44	6.07

Table S6; Related to Figure 5: Top 30 GO categories associated with T0-repressed genes. Extended list of GO categories associated with T0-repressed genes with detailed statistics, corresponding to Figure 5B.

GO biological process complete	# Genes	Fold Enrichment	Bonferroni -adjusted P
positive regulation of T-helper cell differentiation (GO:0045624)	5	22.2	3.3E-02
regulation of p38MAPK cascade (GO:1900744)	6	15.1	3.1E-02
leukocyte cell-cell adhesion (GO:0007159)	7	13.6	9.6E-03
regulation of nitric oxide biosynthetic process (GO:0045428)	8	12.0	4.1E-03
cellular response to chemokine (GO:1990869)	7	11.4	3.0E-02
regulation of interleukin-2 production (GO:0032663)	7	11.4	3.0E-02
negative regulation of cytokine secretion (GO:0050710)	7	11.1	3.7E-02
cellular response to carbohydrate stimulus (GO:0071322)	7	10.9	4.1E-02
positive regulation of T cell mediated immunity (GO:0002711)	8	10.5	1.1E-02
endothelium development (GO:0003158)	9	10.1	3.3E-03
positive regulation of reactive oxygen species metabolic process (GO:2000379)	10	9.0	2.0E-03
granulocyte chemotaxis (GO:0071621)	8	9.0	3.5E-02
positive regulation of myeloid cell differentiation (GO:0045639)	9	8.9	9.1E-03
positive regulation of NF-kappaB transcription factor activity (GO:0051092)	11	8.4	1.0E-03
positive regulation of angiogenesis (GO:0045766)	15	8.1	8.1E-06
positive regulation of lymphocyte proliferation (GO:0050671)	11	7.5	3.0E-03
regulation of response to biotic stimulus (GO:0002831)	11	7.4	3.7E-03
positive regulation of leukocyte migration (GO:0002687)	10	7.0	2.0E-02
cellular response to lipopolysaccharide (GO:0071222)	14	6.8	2.9E-04
regulation of inflammatory response (GO:0050727)	19	5.8	1.4E-05
peptidyl-serine modification (GO:0018209)	11	5.6	4.9E-02
inflammatory response (GO:0006954)	25	5.6	6.4E-08
epithelial cell development (GO:0002064)	12	5.4	2.9E-02
negative regulation of response to external stimulus (GO:0032102)	17	5.1	5.9E-04
cytokine-mediated signaling pathway (GO:0019221)	13	5.0	2.5E-02
negative regulation of transferase activity (GO:0051348)	13	5.0	2.5E-02
regulation of multi-organism process (GO:0043900)	20	4.8	9.1E-05
negative regulation of cell adhesion (GO:0007162)	14	4.8	1.7E-02
wound healing (GO:0042060)	13	4.8	4.0E-02
regulation of MAP kinase activity (GO:0043405)	15	4.8	7.3E-03
